# NF-κB and IRF pathways: cross-regulation on target genes promoter level

**DOI:** 10.1186/s12864-015-1511-7

**Published:** 2015-04-17

**Authors:** Marta Iwanaszko, Marek Kimmel

**Affiliations:** Systems Engineering Group, Silesian University of Technology, Gliwice, Poland; Department of Statistics, Rice University, Houston, TX USA; Department of Preventive Medicine, Northwestern University Feinberg School of Medicine, Chicago, IL USA

**Keywords:** NF-κB, IRF3, Transcription factors, Crosstalk, Innate immune response

## Abstract

**Background:**

The NF-κB and IRF transcription factor families are major players in inflammation and antiviral response and act as two major effectors of the innate immune response (IIR). The regulatory mechanisms of activation of these two pathways and their interactions during the IIR are only partially known.

**Results:**

Our *in silico* findings report that there is cross-regulation between both pathways at the level of gene transcription regulation, mediated by the presence of binding sites for both factors in promoters of genes essential for these pathways. These findings agree with recent experimental data reporting crosstalk between pathways activated by RIG-I and TLR3 receptors in response to pathogens.

**Conclusions:**

We present an extended crosstalk diagram of the IRF - NF-κB pathways. We conclude that members of the NF-κB family may directly impact regulation of IRF family, while IRF members impact regulation of NF-κB family rather indirectly, via other transcription factors such as AP-1 and SP1.

**Electronic supplementary material:**

The online version of this article (doi:10.1186/s12864-015-1511-7) contains supplementary material, which is available to authorized users.

## Background

Identification of pathogen-associated molecules, such as dsRNA and lipopolysaccharide (LPS), by host pattern recognition receptors (PRRs) is a critical step in innate immune response (IIR). Stimulation of TLRs (Toll-Like Receptors) by a pathogen induces activation of signal transduction cascades, which leads to translocation of nuclear factor-κB (NF-κB) to the nucleus [1], activation of interferon regulatory factors 3/7 (IRF3/7) and/or activator protein-1 (AP-1), which cooperate to induce transcription of various cytokines such as alpha/beta interferon (IFN-α/β) to counteract infection [2-4]. In this paper we analyze crosstalk between the two major signaling pathways in the IIR system, namely the NF-κB and IRF pathways. The regulatory mechanisms of activation of these two pathways and their interactions during the IIR are only partially known. Recent work by the Brasier’s group and others has shown that IRF3-dependent and NF-κB-dependent pathways are interconnected at multiple steps, with the final shared component being the IκB kinase-γ (IKKγ) subunit [5,6]. Single-cell imaging experiments have provided information about cellular heterogeneity of these interactions but exact molecular mechanisms are not clear yet [7,8]. In the canonical model, IFN regulation, after RNA virus infection, is conducted by IRF-3 and IRF-7. An explanation was presented by Covert et al. [9] who proposed that the activation of NF-κB by TRIF-dependent pathway is a result of a secondary response to TNFα, which is induced by IRF3 (this latter activated by the TLR4/TRIF-mediated pathway in the first response to LPS) and signals through the TNFα receptor (TNFR)/RIP1 pathway to activate NF-κB. Knowledge about the crosstalk between NF-κB and IRF pathways may be crucial for determining the outcome of viral infection. Most IRF family members are expressed only in specialized cell types, but IRF-3 is widely expressed [10], stimulating synthesis of IFNβ in infected cells. Because of this extensive presence, IRF-3 function is widely targeted by viruses [11], and thus its activity has to be aided by IRF-7 which takes a major part in amplification of the antiviral response. Research on NF-κB-deficient cells has shown that the initial kinetics of the type I interferon (IFN) response depends on concurrent NF-κB activation [12]. Experimental data show that in the absence of NF-κB, the rapid IFNβ expression is blunted, reducing the propagation of anti-viral signals in the mucosal surface [13]. NF-κB also controls expression of the downstream IFN auto-amplification loop through STAT1, IRF-1, −5, and −7 transcription factors.

Taking these findings into consideration it seems logical, that activity of IIR depends on cooperation of both arms of this system and indicate that NF-κB and IRF3 signaling pathways are highly interconnected and that these interconnections influence the kinetics of the IIR [14].

In this paper we examine evidence for a direct crosstalk of the NF-κB and IRF3 signaling pathways at the lowest level, between main transcription factors and genes coding for these transcription factors. Based on experimental data we believe that cross-talk at this level may strongly impact cross-regulation at higher levels. We analyzed respective gene promoters’ sequences using *in silico* methods for identification of transcription binding sites, mainly in the NF-κB- and IRF-coding genes. We cross-reference these *in silico* results with publicly available ChIP-seq data and additional support based on the experimental results from our previous work [13]. Our results extend the results obtained by Brasier’s group.

## Results and discussion

### Interaction between IRF3 and NF-κB Pathways

Using computational methods and cross-species comparisons among human, chimpanzee, mouse and cattle, we analyzed promoters of genes encoding factors involved in IRF and NF-κB pathways. In the first step of analysis we were looking for hypothetical transcription factor binding sites (TFBSs) across given promoter region and in the second step we verified if these TFBS were conserved among species in the conserved domains. Similar method of transcription factor binding sites analysis was used in Iwanaszko et al. [15]. Promoters of downstream genes, mainly coding for transcription factors involved, contain one or more binding sites for IRF and/or NF-κB; partial results are presented in Table 1. Detailed results of analysis of binding to the promoters of the transcription factor genes are presented in Additional file 1. Similar results have been observed for other important genes regulated by NF-κB. The promoter of IFN-β contains NF-κB binding sites and two IFN-stimulated responsive elements (ISREs) recognized by phosphorylated IRF3/7 (data not shown). Previous studies showed that the activity of cooperating regulatory proteins recruited to DNA binding transcription factors play an important role in regulation of gene expression [16-19]. It was already demonstrated [20] that activation of the IP10 but not MCP-1 promoter, both of which contain NF-κB binding sites differing in one and two nucleotides, requires IRF3 as a co-activator following LPS stimulation. This suggests that the binding site sequence composition has an influence on the type of cooperative proteins that are recruited to complex with the NF-κB dimer. Formerly it was also shown that the glucocorticoid receptors can selectively trans-repress the transcription of a subset of genes (such as Scyb9), with promoters which use IRF3 as an essential co-activator of NF-κB binding upon LPS stimulation [21]. This indicates that binding sites arrangement and possible co-activators/co-repressors are critical for gene expression and thus this knowledge grants a deeper insight into the IRF and NF-κB cross-regulation. It is known that TFBS found using computational methods may be non-functional, and therefore we cross-referenced our results with publicly available ChIP-seq data for IRF3, IRF1 and NF-κB.

**Table 1:** **Summary of TFBS counts in dataset**

**Gene**	**Species**	**IRF family**	**of which IRF3**	**NF-κB family**	**of which REL**
IRF1	Human (var*)	1	0	37	13
	Mouse (var)	0	0	23	8
	Chimpanzee	1	0	37	13
	Cattle	2	0	40	14
IRF2	**Human**	7	**2**	12	6
	**Mouse (var)**	8	**2**	24	8
	Chimpanzee	4	1	10	7
	**Cattle**	8	**2**	21	7
IRF3	Human (var)	1	1	5	4
	Mouse (var)	2	1	17	9
	Chimpanzee	2	1	5	4
	Cattle	2	1	12	5
NFKB1	**Human**	6	**2**	21	6
	Mouse (var)	1	0	3	2
	**Chimpanzee**	5	**2**	19	5
	Cattle	5	0	2	2
NFKB2	Human (var)	0	0	24	8
	Mouse (var)	0	0	24	8
	Chimpanzee	0	0	25	8
	Cattle	0	0	27	9
RELA	Human	1	0	18	6
	Mouse	2	1	14	6
	Chimpanzee	1	0	6	2
	Cattle	0	0	8	4
REL	Human	1	0	28	5
	Mouse	0	0	28	7
	Chimpanzee	1	0	21	3
	Cattle	7	0	4	1

Summary of the counts of TFBS corresponding to the members of IRF and NF-κB families of transcription factors found in the promoters of presented genes in four species: human, mouse chimpanzee and cattle. Motifs overlap; numbers in bold correspond to promoters containing 2 or more IRF3 motifs. Detailed results are presented in Additional file 1. *var – other variants of the promoter exist.

### IRF Family

In the IRF3 gene, we can distinguish 3 variants of promoters in the human and mouse genomes. In humans two of these variants have a single binding site for IRF3 and IRF1, and at the same time have a higher number of NF-κB TFBSs (4 for c-Rel, 1 for NF-κB). Two variants of promoters are placed on the negative strand, adjacent to the part covering 1st and 2nd intron and adjacent exons of gene BCL2L12. In the human-chimpanzee comparison, one IRF TFBS is conserved. There are no IRF binding sites conserved in human-mouse or human –cattle comparisons.

In the IRF1 gene there are no conserved binding sites for IRF but a good conservation of NF-κB binding sites was detected. Interestingly, human IRF1 gene has the highest count of TFBS for p50 and p65 subunit in the dataset. Analysis of single promoter sequences shows that the IRF1 gene has binding sites only for the IRF1 and none for the other IRF family members. This may be an example of autoregulation feedback aided by other transcription agents, such as SP1 and AP-1 or even by members of the NF-κB family.

In IRF2 there is no conservation between human and chimpanzee, but there is a good conservation of TFBS for human-mouse and human-cow comparison (conserved sites for IRF1, IRF2 and IRF3, as well as for the NF-κB family members). Analyzing activity of the IRF family members on their coding genes suggests that regulation is connected with IRF1 activity, based on the number and type of TFBS in promoter region of IRF coding genes, while IRF2 does not take any direct part in regulation of IRF genes. Another conclusion is that regulation of the IRF genes appears to be more sensitive to the direct NF-κB binding than to the IRFs binding, yet unknown transcription factors could be involved. This conclusion is based on the number of TFBS that have been found in the promoters of genes from both transcription factor families.

### NF-κB Family

On the human NFKB1 gene promoter, we have found 2 TFBS for the IRF family members: one overlapping site for IRF1/IRF3 and second overlapping binding site for the IRF1/NF-κB family. In chimpanzee we have found 4 TFBS for IRF family members, two of them were overlapping IRF1/IRF3 binding sites. Only one IRF1/IRF3 site is conserved between human and chimpanzee, there is no conservation with cattle (4 sites for IRF1, no IRF3 and IRF2) or mouse (1 site for IRF1, 1 for IRF3, no IRF2).

For the NFKB2 promoter, we did not find any IRF family TFBSs in all species, apart from 1 weak binding site for the IRF3 in 2 of 4 promoter variants in mouse, overlapping stronger the NF-κB family binding sites.

For the RELA gene, a single IRF1 TFBS was found in the human promoter, however there is no TFBS for IRF2 or IRF3. No IRF binding sites were found in the cattle RELA gene, but 1 TFBS was found in chimpanzee, and two in the mouse RELA gene. None of those were evolutionarily conserved.

There is one IRF1 TFBS in the REL gene, but high number of TFBS for the NF-κB family members. High number of IRF1 TFBS was found only in cattle promoter (6), and none for IRF3.

In the NFKBIA (IκBα) promoter, we have found binding sites primarily for IRF1: 2 in humans, 2 in cattle, 3 in mouse and one in chimpanzee. In human and mouse one of IRF1 TFBS overlaps with weaker IRF3 binding motif. NFKBIA gene promoter contains a high number of NF-κB family binding sites, which are not adjacent to the sparsely distributed IRF binding sites.

Analysis of the promoter region in the NFKBIE (IκBε) gene showed similarly moderate counts of the IRF and NF-κB TFBS, with strong overlapping IRF1/IRF2/IRF3 sites in human, chimpanzee and cattle. In the mouse gene we have found only one binding site for IRF3. Only this gene shows higher than usual count of IRF3 binding sites.

### Analysis of 3’UTR Region

In previous research [22] IRF1 gene was grouped as one of the NF-κB-dependent genes, and according to dynamics of gene expression, as an “Early” gene. At the promoter sequence level it can be clearly seen in our data, based on the number of TFBS for NF-κB family. Taking this into consideration we adopted approach presented in Iwanaszko et al. [15] to analyze the 3’UTR regions of the IRF coding genes, in order to look for possible sequence characteristics similar to those of the NF-κB transcription factor coding genes. Analysis of the 3’UTR regions of human IRF coding genes shows, that the IRF1 3’UTR is the longest one with more than 2000 bp and contains one motif of the ARE class II, 4 motifs of the ARE class I and 8 sequences categorized as the ARE class III, with AT-content around 50%. This is consistent with characteristics of the Early NF-κB-dependent genes.

IRF2 3’UTR region is in the 1000 bp range with 9 sequences categorized as ARE class III and only one ARE I, with AT content around 60%.

The 3’UTR sequence for IRF3 is strikingly different from other two, with only 89 bp length and 42.50% AT content, and no ARE elements present. This may suggest that the IRF3 transcript is very stable. Taking into consideration the promoter and 3’UTR characteristics it is possible that IRF2 and IRF3 are also highly responsive to the NF-κB activity.

### Possible cofactors

When we analyzed the promoters of the genes coding for IRFs and the NF-κB subunits we observed presence of the binding sites for the two other transcription factors, which may take part in the crosstalk between the IRF and NF-κB pathways. One of these transcription factors is AP-1 (JUN), which is active in the TLR signaling pathways and is needed for IFNβ activation [23], and the second one is the SP1, which is reported to act with NF-κB2 subunit in antiviral immune response [24].

Based on TFBS search it seems that AP-1 and SP1 may be regulated by each other. We did not find binding sites for the IRF family members in the AP-1 promoter region except for one IRF1 site. Expression of AP-1 seems to be co-regulated by the NF-κB family members, having binding sites for REL (6 sites), RELA (4 sites) and NFKB1 (4 sites). Regulation of the SP1 expression seems to be independent of direct IRF binding, but may be triggered by the NF-κB. We found 5 theoretical binding sites for the Rel subunit, and one for NF-κB2, which is confirmed to be functional [24]. SP1 was believed to bind sites in GC-rich regions and act as universal activator of housekeeping genes [25], however reports implicate, that SP1 is responsive to intracellular signals. In our dataset GC-content fluctuates around 60%; only the promoters of NFKB2 and NFKBIA genes have less than 55% of GC bases. It is interesting that the IRF genes have rather high GC content in our ranking (with the IRF1 having one of the highest: 64.7%), but do not have the highest count of the SP1 binding sites. It seems that the RELA and RELB genes may be the most the SP1-responsive targets; details are presented in Table 2. To analyze the specificity of presence of AP-1 and SP1 binding sites, we generated a set of 100 random 1 kb sequences, which were analyzed in the same way, as our primary dataset. We compared the average count of TFBSs belonging to AP-1 and SP1 in both datasets, and summary results show that AP-1 seems to be less dataset-specific than SP1 (AP-1DATA = 8.2, AP-1RAND = 9.29; SP1DATA = 7.4, and SP1RAND = 2.4), and thus we propose SP1 as a stronger candidate for a co-factor in the NF-κB/ IRF3 crosstalk.

**Table 2:** **GC content and TFBS for cofactors in dataset**

**Gene name**	**GC %**	**AP-1**	**SP1**
IRF1	64.70	5(5)	11(24)
IRF2	61.50	10(11)	11(25)
IRF3	58.90	5(5)	8(21)
NFKB1	55.50	10(11)	8(17)
NFKB2	51.90	6(7)	9(22)
REL	61.30	4(4)	17(41)
RELA	65.70	5(6)	11(18)
RELB	56.40	9(11)	13(34)

GC content and counts of SP1 and AP-1 binding sites in promoter region of human genes encoding the analyzed transcription factors. Numbers in parentheses are the counts of overlapping motifs.

### Validation of *in silico* findings

In order to increase support for the computationally found binding sites, we performed cross-species comparison and indicated promoter binding regions that were conserved. Additionally we analyzed publicly available ChIP-seq data, to compare *in silico* binding sites with experimental results. We analyzed NF-κB ChIP-seq experiments in 10 cell lines, in one cell line for IRF1, and in 3 cell lines for IRF3, all available under the ENCODE project [26]. Detailed data on cell lines are presented in Methods section. There is only one cell line, GM12878 from blood tissue, in which activity of both transcription factors, NF-κB and IRF3, was analyzed and can be compared. In general, data for NF-κB are consistent among all cell lines considered, and peaks and a strong signal are present in the same regions as those determined computationally, in almost all analyzed cell lines. In case of IRF3 data, the agreement depends on cell line. In particular in HepG cells nearly no binding signal was found for our set of genes. In the promoter of NFKB1 gene very strong and broad peak was found in all cell lines, with the strongest peak centered near TSS (transcription start site) which is consistent across all experiments, and a few smaller peaks which are placed in further parts of promoter region. In summary, the computationally found TFBS are located in the experimentally confirmed binding region. Binding region in the proximity of TSS is also conserved in the species analyzed. In the case of IRF3 binding, we observe a weak binding signal between 450b and 1000b upstream from TSS, what is also consistent with our *in silico* findings. In the dataset for IRF1, a weak binding signal is present across the promoter region and a stronger broad peak is overlapping with the NF-κB binding region in the proximity of TSS. Our computational data found IRF1 TFBS further upstream in the promoter region, but none near TSS.

In the promoter of the NFKB2 gene again we see consistent binding across all cell lines used for NF-κB binding analysis. Shorter gene variants have 2 strong peaks in their promoter region (intron of the longer NFKB2 variant), these regions agree with location of computationally found TFBS for NF-κB family. This binding region overlaps with the strong peak for IRF1 binding, and in these variants we found only one TFBS. In the longer variant our computational analysis shows no TFBS, and ChIP-seq data show binding peak in the region of the first exon of NFKB2, but nearly no signal in the promoter region. There is no binding signal for IRF3 in the promoter region of any variant of this gene which is consistent with lack of computationally found TFBS.

In the RELA gene promoter, a strong binding signal for NF-κB is present in 9 out of 10 cell lines, 5 of which are defined peaks and are mostly consistent with the binding sites computationally located in the region further upstream from TSS. There is also a strong binding signal in IRF3 ChIP-seq data, in all 3 cell lines, which is centered next to the NF-κB binding peaks, and overlapping with even stronger peaks for IRF1. Based on our *in silico* data we did not find binding sites for IRF3 in this region, which is rather well conserved in the analyzed species, with the best conservation between human and cattle in 7 out of 8 human variants, and the worst conservation between chimpanzee and other species (average conservation level 36.57%).

For the REL gene we see peaks in the promoter region in all analyzed NF-κB ChIP-seq data, with strong signal near the TSS and in the (500b - 1000b) upstream region. These regions are overlapped with the strong signal from IRF1 ChIP-seq data, and a negligible signal from one of the IRF3 ChIP-seq experiments. Binding sites in the region close to the TSS are conserved among considered species.

In the promoter region of the IRF1 gene, NF-κB binding sites are well represented by a strong peak near TSS site and by a weaker one in the region (500b - 1000b) upstream of TSS. This is consistent across all cell lines used for NF-κB binding identification and also consistent with our computational data. This region is overlapped by a strong peak signal from IRF1 data and a weak signal from one cell line in IRF3 experiments (GM12878). Again we find conserved NF-κB binding sites in the region close to TSS site. For one longer human variant there is only a strong signal from IRF1 binding.

In the IRF2 gene, ChIP-seq data show peaks in 4 out of 10 NF-κB cell lines; the peaks are centered on the beginning of the first exon and the TSS site. Lower-strength signal is present across the whole promoter region. We found conserved binding sites for NF-κB in the region covered by the ChIP-seq data peak, with conservation across promoter region for human, mouse and cattle of around 61%, while chimpanzee sequence has average similarity of 34% with respect to other species. ChIP-seq data for IRF1 show strong and broad peaks across the whole promoter range and one weak peak for IRF3 in only 1 out of 3 cell lines, which is consistent with our *in silico* findings, and suggests a stronger responsiveness to IRF1.

IRF3 gene promoter shows a moderate response to NF-κB in 4 out of 10 cases, located in the region (0b, 500b) upstream of TSS. Strong peak for IRF1 is located in the same region as the signal for NF-κB binding, overlapping the region of weak signal for IRF3. According to our computational results a strong binding site for IRF1 is present, and binding site for IRF3 is located in the region of low IRF3 binding signal.

Taking into consideration data for all promoters and comparing them with our computational predictions, we conclude that our *in silico* approach has some merit. Computationally found binding sites are predominantly located in regions of experimentally proven bindings and these regions are also considerably well conserved among analyzed species.

## Conclusions

**Figure 1:**
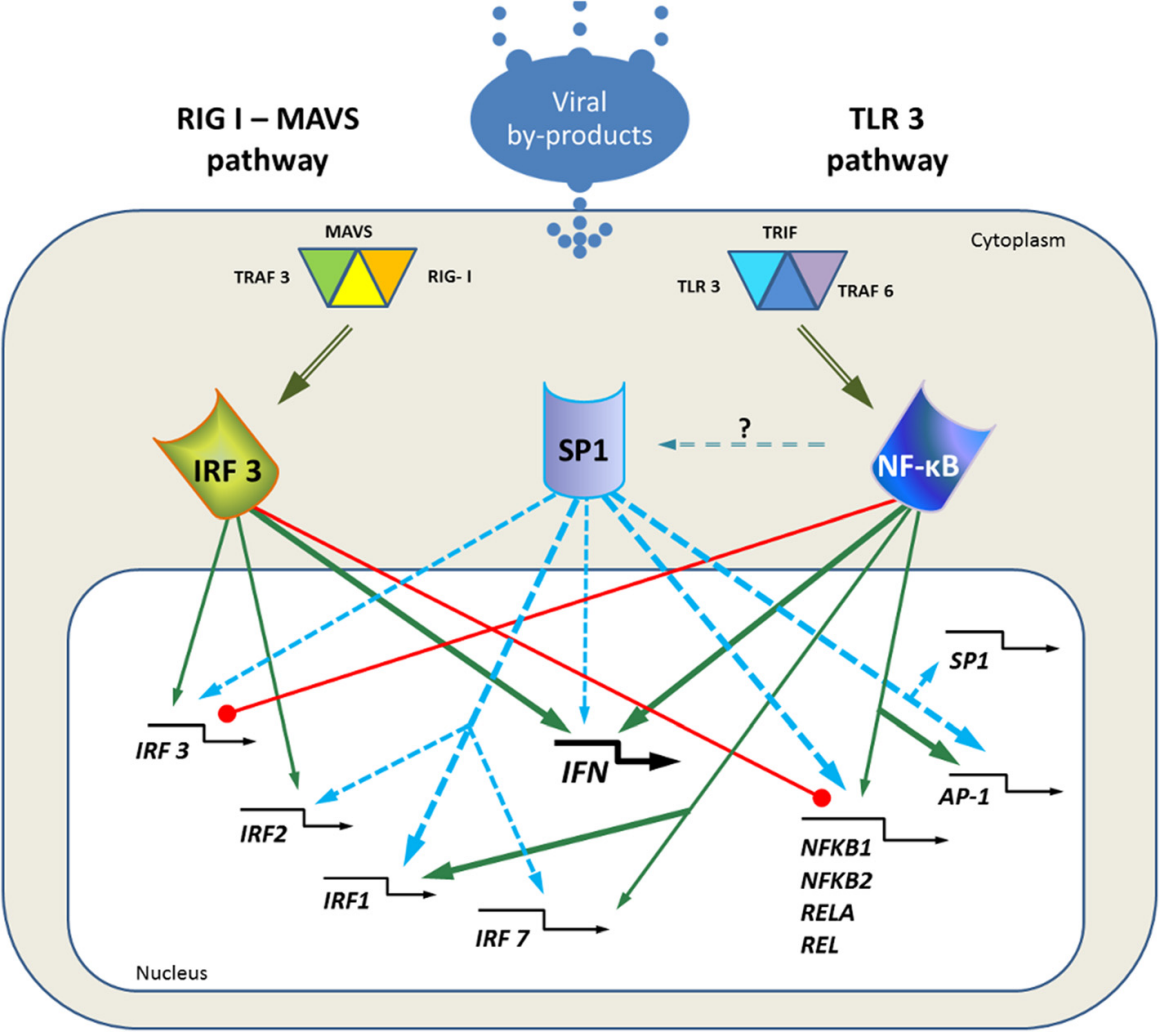
Cross-signaling schematic. Signaling pathways activated by viral by-products and involving potential targets for IRF3, NF-κB and SP1 (including their main target, IFNβ). Green arrows: confirmed positive regulation. Red lines with bullet endings: confirmed negative regulation. Blue dashed arrows: Co-regulation of unknown type inferred from bioinformatics and evolutionary analysis. Bold lines: Strong association of TF based on high counts of binding sites in target gene promoters.

Our cross-regulation hypothesis, based on *in silico* methods is supported by experimental results. Gene knockdown experiments [13] show that levels of RelA are increased by IRF3 siRNA silencing, suggesting that IRF3 may be a negative regulator of RelA expression. In addition, silencing RelA resulted in upregulation of the IRF3 expression levels. Similar results were observed for known NF-κB-dependent genes, such as IL6 or IKBA, expression levels of which were upregulated in response to IRF3 silencing. We show these interactions in Figure 1, which is a proposed conceptual diagram describing interactions between NF-κB and IRF3 pathways at gene promoter level. Presence of interactions between respective transcription factors is based on *in silico* TFBS data with CHIP-seq support as discussed earlier on in the paper, while direction of this interaction is based on literature (green lines) or concluded from experimental data (red lines) [13]. Crosstalk between the two arms of the IIR system, at the level of interactions between genes coding for NF-κB and IRF3 transcription factors and these transcription factors, was not presented before. The results are also consistent with the findings of Wang et al. [27], who show that overexpression of IRF3 in hepatocytes results in IKKβ/NF-κB signaling downregulation [27].

Our results show that binding sites for members of the IRF and NF-κB families do not overlap with each other but tend to be are alternately arranged. The best represented member of the IRF family was IRF1. In most cases only singular IRF3 TFBS were found, usually overlapping the better scored IRF1 binding sites. Compared to the number of the NF-κB binding sites found in the promoter region of genes encoding the members of NF-κB and IRF family, the IRF binding sites are relatively poorly represented. However, location of these few IRF binding sites appears crucial and interrupts proper binding of other (activating) transcription factors. Based on the analysis of TFBS in promoters, the crosstalk between NF-κB and IRF3 pathway is likely biased toward the activity of NF-κB. It was reported that activation of the NF-κB dependent genes occurs with no delays after NF-κB enters the nucleus, whereas IRF3 enters nucleus before NF-κB, but its nuclear translocation profile suggests presence of additional modifying factors, which delay IRF3-mediated activation [28]. We conclude that members of the IRF family may not have a strong direct impact on the regulation of genes encoding the members of the NF-κB family, but rather act indirectly via other transcription factors. This cross regulation may be aided by two other transcription factors, which are distinguished by high counts of their TFBS in our dataset: AP-1, which targets the IRF3 gene as well as the REL, RELA, RELB genes, and potentially SP1, which targets all TF-coding genes in our dataset, and also targets AP-1. SP1 is known to regulate expression of genes involved in apoptosis, cell proliferation, cell differentiation and viral immune response [29]. To obtain a better insight into the relationship between analyzed factors we also examined the promoter regions of the genes encoding the SP1 and AP-1 factors. This analysis shows the presence of crosstalk between IRF3, AP-1, SP1 and members of NF-κB family. These findings are consistent with the results from ChIP-seq data analysis presented in Yang et al. [30], where AP1 and SP1 are spatially oriented relative to the location of the NF-κB family motifs, which suggest that they physically interact. Furthermore observed co-occurrence correlates with different chromatin context [30].

It is also known that IRF1-mediated activation of IL12 needs cooperation with the SP1 binding elements [31]. Taking these data into consideration we present established and hypothetical interdependences in Figure 1. Based on our *in silico* analysis we conclude that SP1 is among the best candidates for the unknown cofactor mediating the crosstalk between the IRF and NF-κB pathways. We present extended crosstalk diagram of the IRF - NF-κB pathways. This work is an extension and a full report of data supporting IIR model in Bertolusso et al. [13], but it is focused on transcriptional regulatory dependencies as well as the role of cofactors SP1 and AP-1 in the cross-talk between NF-κB and IRF.

## Methods

Promoter sequences, defined as 1kB upstream of the TSS, were identified using UCSC Genome Browser [32] and were analyzed using NucleoSeq [33] and ConSite [34] in search for hypothetical transcription factor binding sites (TFBS) specific for members of IRF and NF-κB families. We performed analysis of sequences belonging to human, chimpanzee, cattle and mouse to obtain optimal evolutionary range across mammals; this choice was further discussed in Iwanaszko et al. [15]. Cross-species alignments were performed using ClustalW2 [35]. Cross-species comparisons were further analyzed using ConSite [34] and Toucan3.1 [36]. Binding motifs for the NF-κB family members, as well as AP1, SP-1, IRF1 and IRF2 were identified using position frequency matrices (PFM) available in Jaspar [37] which are the special case of position weight matrices (PWM) used widely for TFBS search. The PFM for IRF3 was obtained from Lin et al. [38]. Consensus sequences for analyzed transcription factors, based on PFM: GAAASSAAANY (IRF3), GAAWNYGAAANY (IRF7), AGGAAATTCCG (canonical RELA), RGGRNNHHYYB (generalized NF-κB). ChIP-seq data were retrieved through the ENCODE project site and the GEO database and used as cross-reference. Sample accession numbers and cell lines of origin can be found in Table 3.

**Table 3:** **ChIP-seq data accession numbers**

**TF**	**Cell line**	**GEO Sample Accession**
IRF1	K562	GSM935546
	K562	GSM935504
	K562	GSM935505
	K562	GSM935549
IRF3	GM12878	GSM935651
	HeLa	GSM935570
	HepG2	GSM935650
NF-κB	GM12878	GSM935478
	GM10847	GSM935527
	GM12891	GSM935526
	GM12892	GSM935285
	GM15510	GSM935529
	GM18505	GSM935282
	GM18526	GSM935281
	GM18951	GSM935531
	GM19099	GSM935273
	GM19193	GSM935279

Accession numbers and cell line origin of data used for cross-reference with *in silico* results.

## Additional files

Additional file 1:
**Full dataset summary.** Table presents the counts of TFBS corresponding to the IRF family and the NF-κB family of transcription factors, found in promoters of presented genes in four species: cattle (bosTau), mouse (mm), chimpanzee (panTro) and human (hg). Yellow rows correspond to promoters with at least one motif for IRF3; orange rows correspond to promoters containing motifs of all members of the IRF family; and rows in bold corresponds to promoter sequences with SP1 TFBS count higher than average (7.4 motifs/promoter) for our dataset.
