# Supplementary material for: NF-κB and IRF pathways: cross-regulation on target genes promoter level
Source: BMC Genomics. 2015 Apr 17;16(1):307. doi: 10.1186/s12864-015-1511-7 (PMC4430024; doi:10.1186/s12864-015-1511-7)
Supplement: Additional file 1: — Full dataset summary. Table presents the counts of TFBS corresponding to the IRF family and the NF-κB family of transcription factors, found in promoters of presented genes in four species: cattle (bosTau), mouse (mm), chimpanzee (panTro) and human (hg). Yellow rows correspond to promoters with at least one motif for IRF3; orange rows correspond to promoters containing motifs of all members of the IRF family; and rows in bold corresponds to promoter sequences with SP1 TFBS count higher than average (7.4 motifs/promoter) for our dataset. [file 12864_2015_1511_MOESM1_ESM.pdf]

Table S1

Table presents counts of TFBS corresponding to IRF family and NF-κB family transcription factors, found in promoters of presented genes in four species: cattle (bosTau), mouse (mm), chimpanzee (panTro) and human (hg). Yellow rows correspond to promoters with at least one motif for IRF3; orange rows correspond to promoters containing motifs of all members of IRF family; rows in bold corresponds to promoter sequences with SP1 TFBS count higher than average (7.4 motifs/promoter) for our dataset.

Query ID	RefSeq ID	IRF1	IRF2	IRF3	IRF7	NF-kappaB	NFKB1	REL	RELA	AP1	SP1
IRF1-b	bosTau7_refGene_NM_001191261	2	0	0	0	5	5	10	7	3	6
IRF1-m	mm9_knownGene_uc007iww.2	0	0	0	0	4	2	7	5	8	4
IRF1-m	mm9_knownGene_uc007iwz.2	0	0	0	0	4	3	7	5	7	4
IRF1-c	panTro gi|291061371	1	0	0	0	4	5	9	6	5	**12**
IRF1-h	hg19_knownGene_uc003kxa.2	1	0	0	0	4	5	9	6	5	**11**
IRF1-h	hg19_knownGene_uc003kxb.2	2	0	0	1	5	2	3	2	7	**12**
IRF1-h	hg19_knownGene_uc010jdt.2	0	0	0	2	2	0	2	1	6	6
**IRF2-b**	**bosTau7_refGene_NM_001205793**	**3**	**1**	**1**	**2**	**5**	**2**	**6**	**2**	**9**	**9**
IRF2-m	mm9_knownGene_uc009lqo.2	3	1	1	1	6	3	6	3	4	**13**
IRF2-m	mm9_knownGene_uc009lqp.1	1	0	0	1	2	0	6	1	12	2
IRF2-c	panTro gi|291061372	2	1	1	0	1	1	7	1	6	2
IRF2-h	hg19_knownGene_uc003iwf.4	2	1	1	2	2	1	4	1	10	**11**
IRF3-b	bosTau7_refGene_NM_001029845	1	0	1	1	3	1	4	2	11	**10**
IRF3-m	mm9_knownGene_uc009gsm.1	1	0	1	1	4	1	5	3	12	5
IRF3-m	mm9_knownGene_uc009gsn.1	1	0	1	1	4	1	5	3	12	5
IRF3-m	mm9_knownGene_uc012fka.1	2	0	1	1	4	1	3	2	9	2
**IRF3-c**	**panTro gi|291061357**	**1**	**0**	**1**	**1**	**1**	**0**	**4**	**0**	**4**	**8**
**IRF3-h**	**hg19_knownGene_uc010end.2**	**0**	**0**	**1**	**0**	**1**	**0**	**4**	**0**	**5**	**8**
**IRF3-h**	**hg19_knownGene_uc002poz.1**	**1**	**0**	**2**	**1**	**1**	**0**	**4**	**0**	**4**	**8**
IRF3-h	hg19_knownGene_uc010ene.1	1	0	0	1	3	2	5	2	4	0
IRF7-mv1	mm10_knownGene_uc009kkg.2	1	0	0	1	3	0	5	3	15	5
IRF7-mv2	mm10_knownGene_uc012fwt.2	1	0	0	2	3	0	4	3	13	6
**IRF7-hv1**	**hg19_knownGene_uc001lqf.3**	**4**	**2**	**2**	**2**	**1**	**2**	**6**	**1**	**6**	**10**
**IRF7-hv2**	**hg19_knownGene_uc001lqg.3**	**5**	**2**	**2**	**2**	**1**	**2**	**5**	**1**	**7**	**10**
**IRF7-hv3**	**hg19_knownGene_uc001lqh.3**	**5**	**2**	**2**	**2**	**0**	**0**	**3**	**0**	**10**	**10**
**IRF7-hv4**	**hg19_knownGene_uc009ycb.3**	**1**	**1**	**1**	**0**	**1**	**2**	**5**	**1**	**7**	**16**
NFKB1-b	bosTau7_refGene_NM_001076409	4	0	0	1	0	0	2	0	11	2
NFKB1-m	mm9_knownGene_uc008rlw.1	1	0	0	1	0	1	2	0	8	3
NFKB1-m	mm9_knownGene_uc008rlx.1	1	0	0	1	4	3	8	3	7	3
NFKB1-m	mm9_knownGene_uc012cye.1	2	0	1	2	3	1	5	3	14	3
NFKB1-m	mm9_knownGene_uc012cyf.1	0	0	0	0	1	2	5	1	14	5
NFKB1-m	mm9_knownGene_uc012cyg.1	1	0	0	0	0	0	5	0	14	3
NFKB1-c	panTro3_xenoRefGene_NM_001165412	2	0	2	2	3	3	3	4	7	5
**NFKB1-h**	**hg19_refGene_NM_001165412**	**3**	**0**	**2**	**3**	**4**	**3**	**3**	**3**	**10**	**8**
**NFKB2-b**	**bosTau7_refGene_NM_001102101**	**0**	**0**	**0**	**0**	**5**	**4**	**5**	**2**	**6**	**10**
**NFKB2-m**	**mm9_knownGene_uc008hst.2**	**0**	**0**	**0**	**0**	**4**	**2**	**6**	**4**	**12**	**10**
**NFKB2-m**	**mm9_knownGene_uc008hsv.1**	**0**	**0**	**0**	**0**	**4**	**2**	**6**	**4**	**12**	**10**
**NFKB2-m**	**mm9_knownGene_uc008hsx.2**	**0**	**0**	**1**	**0**	**6**	**5**	**7**	**4**	**11**	**9**
**NFKB2-m**	**mm9_knownGene_uc008hsy.2**	**0**	**0**	**1**	**0**	**5**	**5**	**7**	**3**	**10**	**15**
**NFKB2-c**	**panTro3_gold_AACZ03073940.1**	**0**	**0**	**0**	**0**	**6**	**4**	**5**	**3**	**6**	**10**
**NFKB2-h**	**hg19_knownGene_uc001kva.3**	**1**	**0**	**0**	**0**	**2**	**6**	**3**	**2**	**7**	**8**
**NFKB2-h**	**hg19_knownGene_uc001kvb.3**	**1**	**0**	**0**	**0**	**4**	**8**	**4**	**4**	**4**	**10**
**NFKB2-h**	**hg19_knownGene_uc001kvd.3**	**0**	**0**	**0**	**0**	**6**	**4**	**5**	**3**	**6**	**10**
**NFKB2-h**	**hg19_knownGene_uc009xxc.3**	**0**	**0**	**0**	**0**	**7**	**5**	**7**	**4**	**6**	**12**
**NFKBIA-b**	**bosTau7_refGene_NM_001045868**	**2**	**1**	**0**	**1**	**6**	**5**	**11**	**4**	**7**	**8**
NFKBIA-m	mm9_refGene_NM_010907	3	0	1	3	6	4	6	5	6	6
NFKBIA-c	panTro gi|291061362	1	0	0	0	1	0	2	0	7	3
NFKBIA-h	hg19_knownGene_uc001wtf.4	2	1	1	3	7	4	5	3	2	7
NFKBIE-b	bosTau7_refGene_NM_001130746	3	1	3	2	5	4	5	3	13	6
NFKBIE-m	mm9_knownGene_uc008cqv.1	0	0	1	1	3	3	4	2	17	5
NFKBIE-c	panTro gi|291061370	5	1	4	4	3	3	3	2	13	5
NFKBIE-h	hg19_knownGene_uc003oxe.1	5	1	4	4	3	3	3	2	13	5
RELA-b	bosTau7_refGene_NM_001080242	0	0	0	1	1	2	3	1	5	6
RELA-m	mm9_refGene_NM_009045	1	0	1	3	3	3	5	2	4	4
RELA-c	panTro3_refGene_NM_001246630	1	0	0	0	1	1	1	2	7	7
**RELA-h**	**hg19_refGene_NM_001145138**	**1**	**0**	**0**	**1**	**4**	**2**	**4**	**3**	**5**	**11**
REL-b	bosTau7_refGene_NM_001192970	0	0	0	0	1	1	1	0	15	1
REL-m	mm9_refGene_NM_009044	0	0	0	2	4	4	5	4	4	7
**REL-c**	**panTro3_gold_AACZ03012369.1**	**0**	**0**	**0**	**1**	**3**	**4**	**2**	**2**	**4**	**15**
**REL-h**	**hg19_refGene_NM_002908**	**0**	**0**	**0**	**1**	**4**	**4**	**3**	**3**	**4**	**17**
